# Therapœia

**Published:** 1825-04-01

**Authors:** 


### THERAPŒIA.

Pharmaca nulla valent, nisi quæ sint commoda causis.

1. *Hæmatemesis cured by Emetics.** The practice of giving emetics in vomiting of blood is not new—but it is very rarely put in force. There is something repugnant to the feelings, or at least to the preconceptions, in giving an emetic to a man vomiting or spitting up quantities of blood. But we are, as yet, very imperfectly acquainted with the modus operandi—or even the *effects* of medicines on the human frame. Many years ago we saw a surgeon, by no means well versed either in therapeutics or pathology, exhibit a scruple of the sulphate of zinc to a man who had vomited up nearly a chamber-potful of blood, and who appeared nearly in articulo mortis. The patient vomited twice or thrice after the medicine was exhibited—but little or no blood appeared in the ejected matters, and he quickly recovered. We confess that we have not had courage enough to pursue the same practice since.

Dr. Sheridan informs us that his father, who practised medicine with repute in the county of Cavan upwards of half a century ago, was in the habit of giving emetics of ipecacuan in cases of hæmatemesis, and that they never disappointed his expectations. Dr. Sheridan, the younger, was deterred from following his father's practice for many years; but having witnessed some fatal cases of the disease in question, he at length ventured on the remedy, and the present paper contains the particulars of five cases successfully treated. We shall give a brief outline of two or three of these cases, as they may inspire confidence in the treatment of a formidable disease.

*Case* 1. Jane Halpen, a young woman, complained of great oppression and pain about the præcordia, attended with violent palpitations. When our author first saw her, she had thrown up much dark-coloured blood: but the vomiting had stopped, though the præcordial oppression continued. He did not, therefore, give the emetic. On the third day, the hæmatemesis returned with violence, and was followed by great debility. On the third day from the last mentioned attack, it recurred, for the third time, and was excessive in quantity. He now exhibited thirty grains of ipecacuan. The vomiting ceased for a few minutes, but soon came on again, and blood was discharged, both grumous and fluid. She now lay in a state of the most alarming debility. No more blood issued—dropsy supervened, and was removed by aperients and diuretics.

* Dr. Edward Sheridan. Dublin Trans. vol. iv.

*Case* 2. A builder, much exposed to atmospheric vicissitudes, had not enjoyed good health for ten years past. He was subject to much pain and oppression about the præcordia. On the 24th December, he was seized with hæmatemesis to a great extent, attended with much palpitation, and pain across the præcordia. He had ejected more than three quarts of blood, when our author ordered an ipecacuan emetic. It rested some minutes on the stomach, and then brought off about a pint of blood, at three or four operations. "Here all the symptoms subsided: he found himself perfectly relieved: heat was gradually diffused over his whole surface, which had been cold before. He fell asleep: a gentle perspiration ensued, which continued a good part of the next day: his pulse became full, soft, and regular, and he felt himself, he said, more free from pain, oppression, or sickness, than he had been for years before. On the day after I gave him a mercurial purge, which brought away a great quantity of such matter as he had vomited, with this difference, that it was blacker, and resembled the discharges in melæna. He continued to improve, but was still weak: in this state, contrary to the most positive injunctions, he made too free, and the pain across the præcordia, the oppression, &c. returned. Under these circumstances I did not hesitate to repeat the ipecacuan: all these symptoms vanished, and he since continues to improve." 46.

*Case* 3. This was a relapse of the first case, after an interval of three years. When our author arrived, the poor woman had vomited more than three quarts of blood. Her anxiety was extreme—pulse scarcely perceptible—palpitation great. With difficulty an emetic was administered. After taking it, her stomach became quiet for ten minutes. She then threw up a quantity of watery and mucous fluid, but no blood. "Immediately after, all the symptoms, as if by magic, ceased."

"These cases," says our author, "with the two others which I observed in the country, have made such an impression on me, that I never can hereafter hesitate for a moment to have recourse to the ipecacuan emetic in hæmatemesis. I do not mean by this term a mere vomiting of blood, which may be produced by various causes, and in some cases of which emetics might prove very prejudicial, but the true hæmatemesis, which I think will always be sufficiently distinguished by the symptoms which have been above described." 48.

---

2. *Hospital Reports.** Authentic hospital reports are always valuable, and Dr. Hosack is a man of talent and observation. This short report comprises only the diseases treated in the New York Hospital, during the months of December, January, and February of 1824.

---

* David Hosack, New York. New York Medical and Physical Journal, No. 9.

We shall give a very concise notice of a few of Dr. H's clinical observations.

1. *Amenorrhœa.* In a case of this disease, of ten years standing, which had resisted the ordinary alvetic and mercurial emenagogues, an injection, per vaginam, (thrice a day) of the aqua ammoniæ diluted with rain water—in the proportion of a drachm of the former, to eight ounces of the latter—excited a slight irritation, and in about five weeks the catamenia were restored.

2. *Intermittent Fevers.* The sulphate of quinine was successful in Dr. Hosack's hands—but more especially the supersulphate, in the following form:—

℞ Sulph. quininæ ʒj.

Acid. sulph. aromat. ʒss.

Syr. Zingiberis ℥ij.

Misce.

Of this mixture a tea-spoonful was given every hour; and, in every case, with the most perfect success.

"3. *Phthisis.* In several cases of this disease, under Dr. Hosack, the most decided benefit was obtained from the tonic treatment, particularly from the bitter infusion in combination with the sulphuric acid, and by the fumes of boiling tar, as recommended by Sir Alexander Crichton."

4. *Chronic Rheumatism.* Against the opprobrium medicorum, Dr. Hosack found more benefit from the tartar emetic ointment, quickened by some of the tinctura lyttæ, than from any other application. With this, the limb or part affected was ordered to be rubbed three times a day. The eruption of pustules rarely failed to relieve the rheumatism.

5. *Syphilis.* Dr. Hosack prefers the oxymuriate of mercury to every other form. It certainly is not so apt to affect the mouth as calomel or the mercurial ointment—nor are patients so liable to catch cold while taking this medicine as they are under other modes of the administration of mercury.

---

3. *Puerperal Peritonitis.* Mr. Davies, in a paper on this subject in the Medical Repository, professes his conviction that the disease is contagious. In the autumn of 1822, he met with twelve cases, while his medical friends in the neighbourhood did not meet with any, "or, at least, very few." He could attribute this circumstance to no other cause than his having been present at the examination, after death, of two cases, some time previous, and of his having imparted the disease to his patients, notwithstanding every precaution.

Out of these twelve patients, three died, "although the antiphlogistic plan, in the form of bleeding, purging, leeching, and blistering, &c. was tried to its full extent." Sedatives also were tried, in every form, with the intention of allaying the pain and the irritability of the system attending the disease—but all in vain.

"I have," says he, "in a number of instances, seen bleeding to its very fullest extent tried, followed by purging with calomel, salts, &c. yet the disease, in seven cases out of ten, gained ground, and ultimately killed the patients. I have also had an opportunity, at different times, of examining fourteen cases after death; and the appearances in all, one excepted, were similar. These appearances were, a high degree of inflammation of the peritoneum, especially that portion of it covering the back part of the uterus and front of the rectum; from a pint to two quarts of sero-purulent fluid in the bag of the peritoneum; an adventitious membrane formed on the surface of the peritoneum, glueing the intestines together in one mass; the substance of the uterus, in some cases, almost in a state of gangrene; sometimes the mucous coat of the intestines inflamed; and the arachnoid membrane of the brain inflamed in some cases."* 184.

Mr. Davies goes on to notice a method of treatment which he has found hitherto invariably successful. He had been in the habit, for some years, of treating common peritonitis, after one good bleeding, with calomel and opium, "given in pretty large doses, and repeated every two or three hours, till the gums became affected." "I can confidently assert," says he, "that since I have used that plan I have only lost one patient. In this case the remedy had no fair trial." He has tried this plan, in puerperal peritonitis, with bleeding first, and without bleeding, and he has found it to answer both ways. "But when bleeding is premised, the gums become sooner affected by the mercury, and the disease, of course, sooner gives way." He has tried this plan of treatment in nine cases successively of puerperal peritonitis, with the best success. In seven of them bleeding was premised, and these appeared to get well sooner than the other two.

"After bleeding pretty largely, five or six grains of calomel, combined with a grain of opium, should be given every three or four hours, till the gums become decidedly affected. The calomel then should be reduced in quantity, or substituted by the blue pill, or, in some cases, left off altogether. The object in view is to keep the system slightly under the use of mercury for a few days. As soon as the pain has given way, and the mouth become a little sore, the patient may take a slight tonic, such as infusion of calombo with a little nitre dissolved in it. The bowels should be kept open by clysters till the mercury has affected the system." 186.

Leeches, blisters, and other auxiliaries, are not to be neglected.

We need hardly say that the plan recommended by Mr. Davies is not new. We should be disposed to recommend it, from some experience

---

* Med. Repos. September, 1824, p. 184.

of its effects, did we not know that, in the present state of feeling—

"To make a mouth sore is voted a bore,"

and that it is considered far more politic to let the lady die with a sweet breath, than have her grumbling for a fortnight that she cannot eat a crust of bread in consequence of the vile mercury.

---

4. *Buchu Leaves.* (Diosma crinata)*. Dr. M'Dowell has recently related some cases of disease of the bladder treated with the remedy at the head of this article. He observes that a chronic form of inflammation very frequently affects the mucous membrane of the bladder, and which, when neglected, extends to the ureters and kidneys, producing a train of severe local, as well as constitutional symptoms. The causes are sometimes obscure. Sometimes they can be traced to mismanaged gonorrhœa—neglected retention of urine—disease of the prostate—stricture of the urethra—or calculous affection.

"The morbid alterations of the mucous surface of the bladder, produced by this disease, are different degrees of vascularity, from merely a few patches of a dark or a bright red colour, to an entire vascularity, in some cases so marked, as to appear as if the bladder had been daubed over with blood; the veins in general are turgid; the membrane becomes much thickened; frequently numerous ulcers form, covered with a tenacious brownish-coloured lymph; sometimes these are very numerous and deep, so as to give a honey-comb appearance to the membrane. The inflammation may run so high as to end in complete sphacelus of the interior of the bladder—I saw this in two instances. The mucous membrane generally forms numerous rugæ, which may be matted together by coagulable lymph.

"The discharges coming from a membrane so altered by disease, are blood, in general venous, and often in very large quantity; a slimy, tenacious mucus; a powdery, white sediment; or a fetid sanious matter.

"The cellular substance under the mucous membrane becomes filled with lymph, and in consequence is liable to be much increased in depth. The muscular fibres are usually much thicker and stronger, and the intervals between them may be filled with lymph: occasionally small abscesses form in the muscular parietes. In one instance I saw an abscess formed between the muscular layer and peritoneal coat, which attained considerable size, and apparently was caused by irritation from a long continued disease of the mucous membrane.

"The constitutional symptoms attendant on the disease are, great derangement of the digestive organs, as indicated by loss of appetite; thirst, often very urgent; tongue white, or loaded with a yellowish brown mucus; nausea, sometimes vomiting; a costive state of the

---

* Dr. M'Dowell. Dublin Transactions, vol. iv.

bowels; fæces usually dark-coloured; a harsh dry skin, and emaciation." 133.

The cases detailed are only three in number.

*Case* 1. Was hopeless apparently. A gentleman had been six years ill—emaciated—debilitated—paralytic of the lower extremities. He passed his urine with great difficulty, being loaded with much tenacious stringy mucus. His appetite was totally gone. After trying several remedies recommended in irritable bladder, he exhibited the infusion of buchu leaves, with tincture of the same and tincture of cubebs thrice a day. In six days the patient's appetite was improved, his strength increased—the mucus in his urine diminished—the ability to retain his water augmented. He continued to improve for a considerable time; but it appears that he relapsed—ostensibly because he could not procure buchu leaves.

*Case* 2. Philip Dwyer, aged 67 years, sallow complexion, emaciated, ill for three years. Complains of severe pain in the pubic region, particularly before he passes water. Great irritability of bladder, passing water in small quantities every quarter or half hour during the night: during the day can occasionally retain it for two hours. Less irritability when using much walking exercise; when sitting is affected with a stinging or scalding sensation in the prostatic region. Urine generally white or muddy. Frequently passes a large quantity of a slimy, pale, yellow-coloured mucus, voided with great difficulty, and soon putrefying: is much relieved by its expulsion from the bladder. Is greatly debilitated, and has lost much weight. Tongue loaded with yellowish mucus. Thirst. No appetite. Bowels generally constipated, stools black. No enlargement of the prostate could be felt.

The disease had commenced three years previously. The following mixture was ordered.

℞ Infus. buchu ℥vij.

Tinct. ejusdem.

——— cubebæ āā ℥j.

Capiat ℥j ter in die. This was continued from the 20th May till the 4th August, when he reported himself as able to follow his daily avocation of a labourer.

We have never employed this medicine. But we are acquainted with a very old gentleman who has had catarrhus vesicæ for many years, and who assures us that he has derived great benefit from what he calls his buchu tea. In a disease so troublesome and difficult of cure, the remedy is worth trial.

---

5. *Tartar Emetic Ointment in Epilepsy.* In the 4th volume of the Dublin Transactions, Mr. John Creighton has detailed six cases of epilepsy relieved greatly, though not cured, by tartar emetic ointment.

Into the Foundling Hospital of Dublin many children are admitted who are affected with epilepsy. This excited the attention of the medical attendants, and some of the worst cases were subjected to the treatment in question. Two of the cases had been rendered perfect idiots by the frequent recurrence of the paroxysms, and others laboured under such aberration of mind as to be but a few degrees removed from the same degraded state. Although a complete cure was, in no instance, effected, " yet we have, says Mr. C. the satisfaction to state that the fits are now, in each patient, comparatively of rare occurrence, and are of so mild a character, as not to interfere with the health or strength of the individual." Indeed, these hitherto helpless objects are now capable of applying themselves to various useful employments, and are no longer a burthen to the institution.

Each patient was allowed to remain a certain time in the infirmary before the use of the ointment was commenced, both to enable the medical officers to ascertain the time of recurrence and duration of each fit, and also to submit them to an antiphlogistic regimen. This last had no influence whatever on the disease itself, and was suggested merely from the plethoric habit of body which appeared in all of them on first coming into the infirmary. The eruption varied much as to its first appearance, sometimes coming forth in 24 hours, sometimes not for three or four days. The eruption was not confined to the part where the ointment was rubbed. Our author says that, "*it most frequently appears in very remote parts.*" From having used the ointment in some hundred instances, we can confidently aver that it does not appear remotely in one instance in ten. The cases of epilepsy we need not detail. It is remarked, in conclusion, by Mr. Creighton, that the tartar emetic ointment has been used at the Foundling Hospital in every case of hooping cough, during the last year, with evident good effect. The paroxysms were diminished not only in violence and frequency, but the progress of the disease so much shortened that, in no instance, did it continue more than three weeks. The application of the ointment was along the spine.

---

6. *Nitre extracted from the Stomach.* Mr. Ferrall (of Dublin) was called to a gentleman who had, by mistake, swallowed rather more than an ounce of nitre. Mr. F. took with him Mr. Jukes's apparatus, and arrived at the patient's house 20 minutes after the accident. The patient was pale, and covered with cold sweat—complained of painful sense of heat along the centre of the chest to the stomach, with a constriction of breathing. He was directed to drink a large quantity of sugar and water, while the surgeon was preparing the instrument. The point of the flexible tube was then passed cautiously between the velum pendulum palati and base of the tongue till it reached the posterior wall of the pharynx. On pressing the instrument down through the œsophagus, the surrounding muscles were thrown into strong action, but this was steadily overcome. When the operator was preparing to exhaust, a violent paroxysm of coughing was induced by the pressure of the tube on the glottis or epiglottis. It was, therefore, kept raised above and behind that sensible part, and the contents of the stomach were speedily drawn off. He then disengaged the syringe, filled it with sugar and water, and injected its contents into the stomach, evacuating it again by the same channel. The patient was fatigued, but not so much so as after the operation of an emetic. He experienced no further inconvenience except a slight rawness in the throat, and speedily recovered.—*Med. Repos. Sept.*

---

7. *Sulphate of Quinine.** The efficacy of this remedy, and its great superiority over every other form of bark, are now almost beyond a doubt. Dr. Barker, while in attendance at Sir Patrick Dunn's hospital, took an opportunity of ascertaining the power of sulphate of quinine in curing intermittent fever, and has presented us with a synoptical table of the results in thirty cases of the disease. Of these thirty cases, not one resisted the remedy. In a majority of instances, the paroxysms ceased within a day or two after the first dose was taken. As a part of the object was to determine the smallest quantity capable of effecting a cure, very small doses were employed in many cases. Although the point is not completely ascertained, yet it was, at least, proved that a grain or less, taken three or four times a day, was as efficacious as larger doses. In one instance, half a grain three times a day suspended the paroxysms for eight days. In no case did it disagree with the stomach. Rejecting a few cases in the table, in which a large quantity of the sulphate was given, we find the average quantity to amount to somewhat more than nine grains. As it is of much consequence to the practitioner to ascertain that this medicine is genuine, we shall subjoin the following characteristics, as drawn up by Dr. Barker:

"When exposed to heat on a slip of platina foil, it melts like wax, it then blackens, partly rises, and burns, with flame. It requires at least three hundred times its weight of water for solution; much more of it is taken up by hot than by cold water, from which it crystallizes in prisms. It is much more soluble in alcohol than in water, dissolving in a quantity of rectified spirit, of spec. grav. 840, amounting to about forty times its weight. Its aqueous solution is decomposed by several reagents. Soluble barytic salts, as might be expected, render it turbid; but I have not observed any reagent to produce so striking an effect as *iodine*. I have found a very minute quantity of this substance in water; for example, a grain of iodine heated in a drachm or two of water, to produce in the watery solution of the sulphate of quina, a copious precipitate of a cinnamon brown colour. This precipitate dissolves on heating the liquor: it is also soluble in rectified spirit, and is again thrown down by water. The tincture of iodine may be also applied

---

* Dr. Francis Barker. Dublin Trans. vol. IV.

as a test of the sulphate of quina; this tincture is partly decomposed by water, but the colour of the precipitate is quite different from that produced by the sulphate of quina, which in colour very much resembles the Peruvian bark.

To the above may be added its intensely bitter taste. But we would advise that the medicine be procured from none but the most respectable chemists, as the temptation to adulterate must be considerable on account of its price." 272.

---

8. *Constipation.** There are few medical men unaware of the bad effects which generally result from habitual constipation. But there are comparatively few who know to what extent accumulations of fæcal matters will take place in the colon, and how long they will remain there unsuspected by either patient or practitioner. The two cases related by Dr. Borthwick may be taken as illustrations.

1. The first was an English lady who, three years previously, had been sent to Madeira for incipient phthisis, having severe pain in both sides, cough, dyspnœa, and emaciation. After a year's residence at Madeira, these symptoms vanished, and she returned home apparently cured. Soon after her return to England, where she married, she was assailed by the same train of symptoms which had forced her to Madeira before. At this period, Dr. B. was consulted, and, on a *primâ facie* inspection, put down phthisis in his own mind, as the character of the disease. On a more minute examination, he was gratified to find the pulse only 76—the eye tinged yellow—the tongue loaded—no appetite—bitter taste in the mouth—bowels constipated—and the *pain* seated in the region of the *caput coli* and also in the sigmoid flexure of the colon. Under these circumstances, it was obvious that the source of the cough was under, rather than above the diaphragm, and his opinion was given accordingly, though not credited by the patient or friends at first. Three days' active purgation unloaded the bowels of prodigious quantities of indurated fæces, when the pectoral symptoms vanished, of course.

2. The second case was also that of a lady, who had long been subject to what she termed cramp in the stomach, which was generally of short duration, though violent in degree. On the present occasion, the attack was of a more obstinate character, and resisted the means usually put in force. Our author found the patient sitting up in bed, with pale countenance, cold clammy sweats, tongue brown and parched, thirst urgent, pulse 80. On examination of the abdomen, the track of the colon loaded with indurated fæces could be plainly felt. The practice was obvious. As soon as the irritability was assuaged by the warm bath, and spirituous fomentations, a dose of castor oil was administered, which passed through, without removing any of the scy-

---

* Dr. Borthwick. Ed. Journal, Jan. 1825.

balæ. It was the same with a dose of salts. Fifteen grains of the compound extract of colocynth were then ordered every three hours, with a wine-glassful of saline aperient in the intervals. This plan soon dislodged the enemy in the shape of "an incredible discharge of large hardened lumps," when the "cramp in the stomach" was at an end.

We would advise the young practitioner, when he meets with cases of this kind, to *first* administer a dose of the purgative in conjunction with some opium and hyoscyamus, in order to take off spasmodic strictures from the colon—*then* to purge as actively as he pleases.

---

9. *Severe Affection of the Head.** The subject of this communication was a young lady, ten years of age, and previously healthy, who was seized with head-ache, vomiting, and febrile symptoms, in the latter end of September, 1821. Measles were suspected, and antiphlogistic measures were employed. On the 7th of October, strabismus was observed—on the 8th, a universal spasm came on—the muscles of the face were all in unnatural action—the eyes turned inwards—the angles of the mouth drawn alternately on either side—the muscles of the trunk, and extremities convulsed—the child herself insensible, and resembling a person in a protracted paroxysm of epilepsy. "Both temporal arteries were opened without loss of time; after some blood had flowed, the appearance of the eyes became natural, and the little patient became sensible, and complained of pain in her head. Twelve leeches were next applied to the neck, the head shaved, and subsequently blistered, whilst powders with calomel and scammony were given at intervals, until stools were procured. The pulse at the commencement was 120; it was reduced by the abstraction of blood, but rose again in the evening, when ℥vi. more of blood were taken from the arm; the pulse was again reduced, and the head-ache removed." On the 9th, terebinthinate glysters were employed. In the evening, the head-ache returned, with increase of fever, and constipation of bowels: pulse 120, and full. Six ounces of blood from the arm—a stool procured by purgatives and an enema. 10th. Mercurial ointment to be rubbed on the abdomen thrice a day. 11th. Eyelids convulsed—pulse 120 to 140—frequent sighing. Ten ounces of blood from the arm did not reduce the pulse, but afforded relief to the other symptoms. The blood was buffed and cupped. On the 13th, six ounces of blood were abstracted, and exhibited the cupped and buffy appearance. On the 17th, after a good night, she awoke flushed, with the right eyelid hanging relaxed. Twenty leeches to the temples. 18th. Pulse 100, eyes clear—eye-lids natural. Two large stools—mercurial friction discontinued. From this period she convalesced. She lost in all 50 ounces of blood—took 120 grains of calomel in her purgative powders, and had an ounce of mercurial ointment rubbed in. In six similar cases, which our author had under his charge the result was successful, under a sys-

---

* Dr. John Beatty. Dublin Transactions, Vol. IV.

tem of copious sanguineous depletion. We are ready to agree with our author that "had not a very active practice been employed, hydrocephalic effusion, and consequently a fatal termination, would have been the result." A child of the same family had been carried off the preceding year, by decided hydrocephalus, in whom the disease commenced with precisely similar symptoms.

---

10. *Datura Stramonium.** Little attention was paid, in this country, to this powerful narcotic, till Dr. Mariet's paper on the subject appeared in 1816. Dr. Stork employed it at Vienna, in cases of mania, epilepsy, and convulsive diseases, many years previously. The inhalation of the fumes of stramonium was much employed in this country, some years ago, in spasmodic asthma, and we have seen several instances of its efficacy in this distressing disease. It has also been used a good deal in painful affections of the nerves. Dr. Begbie considers the narcotic properties of the medicine as decidedly superior, in some cases, to those of other medicines of that class. In no case did he perceive any bad effect. A temporary unpleasant nervous sensation in the throat was observed in one case. The form used was Mr. Hudson's extract from the seeds—the dose being $\frac{1}{4}$ of a grain repeated every third or fourth hour during the day. In no instance was the dose raised above half a grain. We shall now proceed to lay before the reader some account of the cases.

*Case* 1. Mrs. W. aged 43, of delicate habit, went to bed, 14th March 1821, in perfect health, but was soon seized with a violent pain in the occiput, followed by flashing of light, tinnitus aurium, and strong pulsation down the neck. When seen, she was pacing the room in great agitation, exclaiming "oh! my head." She soon became faint, sick, and vomited. The surface was cold—the face pale—pulse 80—pupils natural—bowels open. Ten ounces of blood from the arm—a brisk purgative—cold to the head. 15th. Symptoms unabated. A vein opened, but syncope approached, and only six ounces of blood abstracted. The pain and throbbing continued undiminished. In the course of the day, the temporal artery was twice opened, and 24 ounces of blood drawn off, with some relief. 16th. Had a restless night—pain violent all over the head, with strong pulsation at intervals. The complaint continued during the next three days without intermission. From the 19th till the 21st, the pain was principally in the neck, shooting along the whole spine, and attended with strong pulsation. It afterwards affected the upper part of the back, the patient complaining of most painful feelings of anxiety about the chest, with constant and loud yawning, and violent, but not irregular action of the heart. The pain descended, and these symptoms subsided. On the 22d and 23d, she

---

* Cases illustrative of the Sedative Powers of the Datura Stramonium. By James Begbie, M. D. Fellow of the Royal College of Surgeons of Edinburgh.—*Ed. Med-Chir. Trans.*—Vol. 1. p. 285, *et seq.*

screamed violently, from pain in the back of the thigh and leg, extending to the loins and back, attended with frequent slight convulsions of the limbs—the pulse was between 80 and 90, weak. 23d and 24th, had intervals of ease. She chiefly complained of burning heat in both thighs, and a pricking sensation down the limb. These increased till she could not bear the slightest pressure—but had the parts constantly exposed, or covered with iced water. By the 29th, the symptoms had gradually subsided, and there was reason to hope that the disease had run its course. Cold and large doses of opiates were the only remedies from which the patient derived advantage. On the last mentioned date the complaint returned with all its pristine violence, and the usual medicines failed entirely of success. On the 3d April, the extract of stramonium was first tried, in doses of $\frac{1}{4}$ of a grain, repeated every three hours. The first day's administration brought no relief. It was then increased to one third of a grain, and benefit was soon derived from this plan. The patient, however, was obliged to be kept under the influence of the medicine for a fortnight longer, when all symptoms of the complaint were entirely gone, and did not return.

We think the case was decidedly one of hysteria, which will frequently assume even more anomalous forms than the above. It is, however, interesting, independent of its illustration of the power of stramonium.

*Case* 2. W. Balcarras, aged 14, August 1st, 1821, has complained for several days of stiffness and rigidity of the lower extremities, without any assignable cause. These increased, and presently the abdomen became hard and rigid—then the muscles of the neck, back, and jaw—in short, in a few days he exhibited a well marked example of opisthotonos. Bleeding, purging, calomel, opium, and the warm bath, were employed with some relief, but only temporary. The symptoms increased, and on the 6th were at their acmé, the patient resting almost on the occiput and heels, unable to perform the slightest motion for himself—the jaws nearly shut, and fixed immoveably—abdomen as hard as a board—the extremities rigid. Very active measures were resorted to, as bleeding, blistering, sinapisms, opium, calomel, &c. and about the 9th, the symptoms seemed to yield. On the 10th they returned, nearly as bad as ever, with the exception of the trismus, which was, in some measure abated. The stramonium was now given in doses of $\frac{1}{4}$ of a grain three times a day—at first with no apparent benefit; but afterwards, when the dose was increased to one third of a grain, and more frequently repeated, advantage was evidently gained. The disease, however, continued several weeks, but he ultimately recovered in about two months.

*Case* 3. A woman aged 50, had complained for several days, of acute pain between the knee and the foot, deep-seated, and referred to the bone. Various local applications were employed without benefit. The extract of stramonium, in quarter grain doses, every three hours, was recommended; but she left it off before benefit could be reasonably expected. The complaint appeared to give way to large doses of carbonate of iron: but in a second attack, some weeks afterwards, the iron and other remedies failed, and recourse was had to stramonium, in doses of one third of a grain. "The relief was remarkable before many doses were taken; and in the course of a few days the symptoms disappeared."

*Case* 4. This was one of sciatica in a young man, where all the usual remedies proved unavailing. The stramonium was effectual in a few days. Two other cases are related by our author; but they are unsatisfactory.

Perhaps we may be excused for cautioning surgeons against the too free exhibition of this extract, in the form of suppository, in cases of painful diseases of the rectum. An hospital surgeon of celebrity, in this metropolis, prescribed *two drachms* of the extract of stramonium to be made, with some other ingredients, into six suppositories, one to be put up the rectum occasionally when in much pain. One was accordingly introduced. It dissolved, and was entirely absorbed. The patient became affected with symptoms closely resembling delirium tremens, and nearly died. *Sat verbum sapienti.*

---

11. *Peculiar Convulsion in Children.** This is the complaint which we noticed at some length, [p. 452] as described by Drs. Cheyne, Clarke and others.

Mr. Cox, who was apothecary to the Royal Universal Dispensary for Children, has had opportunities of treating several cases of this curious disorder. In respect to the *name*, he would prefer calling it "cerebral croup"—the term *spasmodic croup* having been strongly objected to, "inasmuch as it tends to confound it with cynanche trachealis, from which it differs entirely in its nature." The causes of the disease under review have been considered as various. Thus teething, worms, gastric derangement, have, in their turn, been adduced in the etiological list. What has Mr. Cox to say?

"Cases of this disease have come under my notice where there has not been one symptom of gastric derangement—the belly being free from pain, and the motions perfectly healthy.† On the other hand, I have seen the disease without any symptom of cerebral affection, but with considerable derangement of the abdominal secretions, and sometimes extreme pain in the belly. The disease appears to me to consist of a convulsive action of the trachea, sometimes, but not always, attended with a convulsive action of other parts of the body, as the face, hands, or feet. This morbid action I consider as arising from cerebral irritation, which may be either symptomatic or idiopathic: when it is un-

* Mr. Harry Cox. Med. Repos. February, 1825.
"† Hydrocephalus also sometimes occurs under these circumstances."

attended by symptoms of excitement of the brain, I suppose the diseased action to be transmitted through the medium of this organ, without actual inflammation existing in it. I have known it occur, in three instances, either preceded or followed by mesenteric disease. In the three instances in which I have known it prove fatal, the children all died in a fit which came on unexpectedly. In one of those cases, although the stools had been black, brown, green, and slimy, no structural disease in the abdomen could be detected on a careful dissection: the head was not examined. In another case, the only appearance of disease was ulceration of the internal and middle coats of the small intestines: the head was not examined. I was not present at either of these examinations. Another case was attended with considerable enlargement of the head and the usual symptoms of chronic hydrocephalus: when I last saw this child, I considered it past recovery." 124.

The symptoms of this peculiar affection, we have described, when speaking of Mr. North's paper, and they need not be repeated here. But we shall introduce Mr. Cox's therapeutical observations.

"With respect to the treatment, when it appears to arise from idiopathic cerebral excitement, especially if it be attended with general convulsions, we should lose no time in the application of leeches to the temples and blisters behind the ears. I have put this mode of treatment to the test, by adopting it, and administering no other medicine than the common saline mixture, and with perfect success.*

"* I cannot omit this opportunity of entering my protest against the misplaced timidity with respect to the application of leeches to children. It is said young children cannot bear active or repeated depletion, and that, if submitted to such treatment, they sink under it. This may apply to a child eight days or eight weeks of age, but certainly not to a child of eight months or upwards: after this period they bear depletion well, and possess powers of recovery from an extremity of disease far greater than those of the adult: in the latter there are, in many diseases, symptoms which will induce an experienced Practitioner to say there is no chance of recovery, but will he say so of children? I will venture to assert he will not: and I am sure every mother who has reared a family will bear testimony to the extraordinary powers of recovery which children possess. It is also said that the repeated application of leeches reduces the powers of the system, and renders it irritable, thereby becoming a source of irritation to the existing disease. The soothing plan is at present the favourite with many Practitioners: it has not been my good fortune to witness any good effects from this plan in many cases in which I have seen it recommended; the ill effects arising from it have frequently presented themselves to my notice in the form of chronic structural disease, which admitted of no relief. Within the last six years I have seen upwards of ten thousand cases of diseased children; and, during that time, prescribing, as I have done, for thirty, forty, or fifty children of a morning, I have so constantly been in the habit of seeing neglected cases of inflammation of the lungs or the brain, or of fever accompanied by these, that it is my firm belief more children have died from the neglect of leeching than ever adults have died from famine or the sword."

"Where abdominal irritation appears to be the cause, unattended by any symptoms of disease in the head, we may expect one or other of the following symptoms: either a considerable derangement of the abdominal secretions, or an extremely painful state of the belly, with violent fits of crying and drawing of the legs towards the abdomen, and sometimes the passing of a considerable quantity of wind *per* anum, followed by immediate relief. The secretions of the liver and mucous membrane of the intestines being deranged, the plan I have found most successful is a moderate dose of hyd. submur. and pulv. rhei one morning, and ol. ricini the next, persevered in for some time, giving also twice a day sodæ carb., pulv. rhei, and infus. calumbæ. When pain in the belly exists, it is usually relieved by the warm-bath and a combination of magnes. carb., pulv. rhei, and aq. menthæ. The diet of a child, under these circumstances, is of considerable importance; the breast is by far the best mode of nourishment, and to it the child should be confined, if practicable. If the gums be full, they ought, by all means, to be lanced. The combination of abdominal and cerebral affection is, perhaps, most frequently the case in this disease: when it is so, we should not suppose that mere attention to the abdominal functions will remove the complaint—it will not do so. I recollect, in a case of this description, a friend of mine, who had frequently differed with me in opinion as to the propriety of repeatedly bleeding children, cautioned me against the application of leeches and blisters: it was considered to be a case beyond recovery, there being, to all appearance, effusion in the ventricles. The child was about a year and a half old; and although much emaciated, I applied two leeches to the temple, and rubbed the ung. lyttæ behind the ears: the symptoms were somewhat relieved. The leeches were repeated a second and a third time. The child perfectly recovered; and on being shewn to the gentleman alluded to, he was much surprised. In the course of the disease, the lungs sometimes become the seat of inflammation, requiring the application of leeches to the chest," 125.

Six cases are related by Mr. Cox, in illustration of these precepts; but for these we must refer to the original paper in the Repository.

---

12. *Tobacco in Dysentery.** While exhibiting tobacco in tetanus and epilepsy, Dr. O'Beirne observed that, this agent produced all the effects of copious blood-letting, which effects disappeared in a day or two, leaving the patient as strong as before. He also had seen tobacco, on many occasions, relieve pain and remove constipation. These qualities recommended the remedy, in his mind, for the cure of dysentery. Cases are, therefore, related to illustrate its effects. We shall only give the particulars of one case as a sample.

---

* On the Use and Advantages of Tobacco in the Treatment of Dysentery. By James O'Beirne, M.D. Surgeon-extraordinary to the King, &c. Dublin Institution, Vol. iv. p. 386, *et seq.*

*Case.* Catharine Finlay, ætat. 21, residing in a low swampy situation, was attacked on the 8th September, 1823, with rigors, nausea, griping pains in the bowels, and frequent desire to go to stool, at which times she passed little else than florid blood and mucus. These symptoms continued for three days, without any medicine being taken. 12th. Her mother gave her a dose of castor oil and tincture of senna, which produced a slight feculent discharge, followed by an increase of abdominal pains and urgency to stool. 13th. Our author saw the patient. Her countenance was pale and sunk—tongue white and furred—thirst considerable—skin warm—pain about the umbilicus—tormina—a stool every ten minutes—discharge solely blood and mucus—pulse 100, small and weak.

"An enema, consisting of ten grains of Virginian leaf Tobacco, infused for twenty minutes in six ounces of boiling water, was thrown up, with some pain and difficulty, by a syringe, and as quickly returned without producing any effect. At 4 o'clock P. M. she was directed to take an ounce of oleum ricini, and, in half an hour after, to foment the abdomen with an infusion made by pouring two quarts of boiling water on two ounces of Virginian leaf Tobacco, and allowing it to stand for twenty minutes before use. Directions were given to continue the stuping until giddiness, nausea, and weakness came on, and then to omit it. Nine o'clock P. M. she informed me that the fomentation greatly relieved the tormina and tenesmus; that about half-past 8 o'clock she became very weak; a cold perspiration broke out; she felt her head giddy, and her stomach disposed to turn: that immediately after these symptoms, and just before my arrival, her bowels gave way. On inspection, the discharge was copious, feculent, and mixed with some blood and mucus. Great relief followed; the pulse became soft, full, and 90; the tormina and tenesmus much less urgent; the countenance lost gradually its pale and contracted character; she soon fell into a profound sleep." 389.

From this time she had a rapid convalescence and recovery. Seven cases are related—all evincing the power of the remedy over the disease. The following remarks we shall give in the author's own words.

"On reviewing the foregoing Cases, certain of the effects of Tobacco in Dysentery cannot have failed to fix attention—its power of controlling the undue action of the heart and arteries, and of altering the character of the pulse; the arrest it, as it were, placed on the flow of blood, and on the secretion of mucus from the tract of the digestive tube—the certainty with which it induced that degree of debility necessary to overcome spasm, and favour the unlocking of the bowels—the unerring relief it gave to those obstinate and distressing symptoms, the tormina and tenesmus—the removal of that apparent, not real, debility, which so strongly marks enteric inflammation, and of which paleness of the countenance forms so striking a feature—its rapidly restoring the skin, stomach, and kidneys, to their healthy functions—lastly, its procuring sound sleep, the want of which is so constant a symptom in this disease, and which Rouppe, Pringle, and others, could not induce by opium and the strongest narcotics—these are prominent features of its full influence, and must be recognised in the progress of the Cases just detailed." 402.

Our author confines the administration of the remedy to the form of fomentation alone. He observed, that when the full influence of tobacco is obtained, the abdominal muscles and the muscular coat of the intestines lose much of their expulsive power. This induced him to premise the use of a mild purgative, so as to allow sufficient time for its passage through the stomach, before tobacco was employed, or its effects on that organ exerted. The changes effected by tobacco, according to our author's belief, are, first, a suppression of the inflamed state of the mucous membrane of the intestines—and secondly, a removal of that spasmodic action of their muscular coat, which always accompanies such a state, and on which retention of the feculent contents seems to depend. "Perhaps, therefore, it will be found a good general rule to persevere in the use of purgatives, with the tobacco fomentation, until perfectly natural and feculent discharges be permanently established."

These cases and observations of Dr. O'Beirne are certainly deserving the attention of the profession.

---

13. *Hydrophobia.** We have a *hundred* vaunted specifics for preventing hydrophobia—and only *one* for preventing variola. Yet it will be readily granted that we have more success with vaccination than with all the boasted anti-hydrophobics which have, from time to time, been proclaimed. We fear there is no certain prevention of this dreadful disease but early excision of the parts to which the poison has been applied. But this cannot always be done; as in the following case, for example, recorded by M. Recamier.

*Case* 1. On the 27th of February, 1823, Charles Mignot, a vine-dresser, was assailed by a wolf, whom the courageous young man attempted to seize by the tongue, with the hope of choaking him. He paid dearly for his temerity; for he had his arm torn and mashed in a terrible manner.—Besides this, he received several other severe wounds in his contest with the furious animal. The medical man (M. Barrau) who first saw him, could do little more than cleanse and dress the wounds—since cauterization of so many and such deep injuries was impossible. There was also no certainty that the wolf was mad. On the third day, a copious nasal hæmorrhage took place, and this discharge recurred several times afterwards. By low diet, and great care, all went on well till the 1st of April (M. Recamier has strangely enough made it the 1st of March, which is ridiculous as well as incorrect) when the wounds in the arm and hand were completely cicatrized. At this

---

* Considerations sur la Rage. Dr. Fiévée 8vo, Paris, 1824. D'Hydrophobie. Par le Professeur Recamier.—*Revue Medicale. Aout*, 1824.

time, a severe inflammation occurred in the other hand, though it had not been wounded. This was relieved by leeches and other means. On the night of the 9th April, the patient was seized with cephalalgia of one side, and sense of constriction in the throat. The patient lost his appetite, became morose, fearful of death and had difficulty in swallowing drink, but without any dread of liquids. Pills of assafœtida, musk, and opium. He had some sleep in the beginning of the night, but got up at five in the morning, and went from the house where he lived to that of his mother, in a state of great agitation. M. Barrau this day examined the patient's mouth carefully, and could discover no trace of pustules under the tongue. The difficulty of swallowing still continued. The wounds not yet healed, but looked well. The patient, who was naturally of a good temper, now appeared irascible, and ill-natured. Venesection—increase of the medicines abovementioned—mercurial frictions—exposure to the open air—and every care taken to amuse his mind. The sense of constriction in the throat being great, leeches were applied to that part. 11th. Passed a restless night. He this day put his feet in warm water, without any reluctance; but immediately complained that he felt as if a bar was put across his chest, and threatened suffocation. He occasionally fell into a slumber this day, waking in great fright. 13th. A remission of all the symptoms. The patient ate a soup much better than he had yet done. He also took a warm bath without difficulty. He assured his physician that the constriction of the throat was entirely gone. The mercurial frictions were discontinued as the patient was now in a state of copious salivation. The wounds, yet open, were dressed with mercurial ointment. In the night he had no sleep, and was restless and uneasy all the next day. 15th. The ptyalism is considerably diminished. The patient took some soup, and also some bread soaked in wine, without difficulty. At nine o'clock, he went into the bath, without hesitation, and there fell asleep. He awoke raving, and imagining himself pursued by wolves. 16th. Had some healthy motions this morning—much prostration of strength—acceleration of pulse. Took some soup, and bread soaked in wine, without difficulty. 17th. Had a bad night, with much incoherence of ideas, amounting to delirium. Vomitings of yellow and green bile to day, and expelled a lumbricoid worm. Could not swallow water to aid the vomiting; but afterwards took two glasses of wine, without much difficulty. The prostration of strength increasing—delirium—quick pulse. 18th. Vomitings distressing—augmentation of the delirium—cries, swears, thinks he is pursued by dogs and wolves; total incoherence of ideas—spasms of the members. This state continued till the 20th—when death put a period to his sufferings. The body was not examined.

We think it is evident that either the disease in question was not hydrophobia—or, if it was that dreadful malady, it was greatly modified by the man's constitution, or the remedies employed. It will be remarked that there was a signal mitigation of the symptoms, at the period when the mercurial ptyalism was developed—indeed, there were reasonable grounds of hope that recovery would take place. Had the salivation any share in the mitigation or procrastination of the disease? We are, upon the whole, inclined to doubt the existence of real rabies canina in this case, notwithstanding the authority of Recamier to the contrary. Probably the reader will be disposed to entertain similar doubts respecting the hydrophobic nature of the following case, related by M. Fiévée.

*Case* 2. Madam N——— residing in Paris, aged 46 years, of nervous temperament, sombre and melancholy disposition, was bitten by her own lap-dog in the upper lip, on the 15th June, 1823. The dog bit several other dogs, who all went mad. The dog himself was taken to the "Combat des Animaux," where he also died hydrophobic. The lady, not suspecting, at first, that her dog was mad, took no preventive means; but, after a few days, she went to the place where her dog had been confined, and learnt, to her horror and amazement, that he was dead of hydrophobia. She went instantly to M. Breschet, who cauterized the wound on her lip, with butter of antimony. M. Fiévée was consulted on the fourth day of the accident, and found the lady in great mental agitation respecting the bite. The symptoms, however, were such as might be expected from fright working on the imagination of a nervous and hypochondriacal female. Presently the pulse rose, the skin increased in temperature, and she complained of a constriction about her throat, and also at the epigastrium. She was constantly spitting—sleep forsook her—the appetite failed. As our author apprehended approaching hydrophobia, he persuaded the patient to retire to the village of Vauvres, under the care of Dr. Joanin, of that place, where she was also visited by Messrs. Voisin and Falret, who have their Lunatic Asylum established there. On the 6th day, she was found to be very feverish, with great agitation, amounting almost to convulsions, constantly spitting, thirst moderate. Till the 9th day, there were no other means used than warm baths to the lower parts of the body, refrigerants to the head, and diluents internally. On the 7th, 8th, and 9th days, the physicians in attendance discovered the pustules, described by the Russian physicians, under the tongue of their patient. They disappeared on the 9th, and were not cauterized. As the symptoms were now increasing, the therapeutic agents were also increased. In addition to other means, *fifteen grains* of calomel were given every night, with a grain of the extract of belladonna. From the 18th to the 30th day from the accident, the symptoms gradually diminished—but from the 30th to the 40th day, things retrograded, and apprehensions were entertained that hydrophobia was at hand. It is curious that, at this epoch, the dogs bitten by the lady's lap-dog all died at the "Combat des Animaux," where they had been placed for security and observation. The change for the worse, in the lady's symptoms, induced the physicians to exhibit the oxymuriate of mercury, instead of the calomel—to which was added a combination of sulphate of quinine and opium. These medicines were augmented daily, till at length they were given in very large doses. By these means the lady got well, and returned to her friends.

The history of this case is the most confused and unsatisfactory we have ever read. No mention is made of any mercurialization of the system, although the lady was taking mercury in enormous doses, for weeks together! In short, we can hardly believe the patient actually received the hydrophobic poison—or, if she did, it never affected the system. Whether the mercury, in this case, proved prophylactic, we cannot say. The circumstances are such as to afford a presumption that it had this effect. It goes to corroborate the statements made a few years ago, by Mr. Daniel Johnson, of Torrington, Devonshire, who asserts that mercury *prevented* hydrophobia in every case where it was taken for a certain time after the bite of rabid animals.

---

14. *Compression in Ascites.** Dr. Godelle was led to compression of the abdomen as a mean of curing ascites, from observing that it prevented, at least for a time, the re-accumulation of fluid in the cavity of the peritoneum, after the operation of paracentesis. The following is the case brought forward in illustration of this anti-hydropic agent.

Vorgier, a shoemaker, 18 years of age, of feeble constitution, and subject to nasal hæmorrhages, had the natural small-pox, and afterwards the measles, in the beginning of 1823. On the 15th of April, 1824, after a long and fatiguing journey, on a cold and dry day, he found himself unwell, and affected with lancinating pains at the pit of the stomach, and over the abdomen. Diarrhœa succeeded, and afterwards dysury. He neglected himself, and the diarrhœa continued, attended with so much debility, that he was obliged to seek refuge in the HOTEL DIEU, on the 5th of July. At this period he had fever, vague darting pains at the epigastrium, painful tension of the belly, thirst, high-coloured and scanty urine, dry burning skin, small quick pulse, frequent cough, without expectoration. There was obscure fluctuation on percussion of the abdomen.

It was pronounced that the patient had chronic peritonitis, with incipient effusion. Leeches to the epigastrium and hypochondria—nitrous mucilaginous drink—fomentations—pediluvia, &c. The patient had some appetite. These means were varied or reiterated, according to circumstances, for fifteen days. He could not bear digitalis. The effusion increased daily. He left the hospital on the 21st July—but returned on the 3rd of August, in a still worse condition. The countenance was altered—cough constant—belly swelled, tense, and painful—urine scanty and red—skin dry—diarrhœa—no appetite. Diuretics and other means were tried, but the malady increased, and the abdominal swelling incommoded the breathing so much that the surgeon was on the point of performing paracentesis, when Dr. Godelle thought he would give compression a trial. A bandage was first ap-

* Dr. Godelle. Nouvelle Bibliotheque, Sept. 1824.

plied, but that was always slipping. A broad belt, with lacings like ladies' stays, was next employed, and answered well. Instead of being incommoded by the pressure, the patient breathed easier, and had less pain.* Pressure was made on the 15th, and by the next day, the belly had sensibly diminished. This diminution daily made progress, and by the 20th, or fifth day from its first application, the patient's abdomen was reduced to its natural size, without exhibiting any symptoms of fluctuation. It is proper to state, however, that during this time, digitalis was given in doses of *four grains daily.* "Under the double influence of these agents, the urine flowed in enormous quantities." We leave our readers to determine which of these agents was the *diuretic* on this occasion; for no one will doubt that the prodigious flow of urine was the real and efficient cause of the sudden abdominal diminution.

---

15. *Hydrocephalus cured by Mercury.*† This was a boy, seven years of age, who, for some time previous to application, had been listless and languid—sometimes flushed, sometimes pallid, with thirst, dry skin, and irregular bowels. A physician prescribed some opening medicine, containing calomel, which afforded temporary relief. He afterwards began to complain of dull pain in the forehead, vertex, and sinciput, with pyrexia, all which gained upon him daily. On the 7th day of his illness, Drs. Fisher and Munkittreck were called in, and found the little patient complaining of his head, with ferretty eyes, pulse 110, and firm, tongue loaded, bowels very irregular, evacuations alternately greenish, slimy, and mucous.

The bowels having been repeatedly evacuated, our authors were determined to attend to the head. This was shaved—cold washes applied—twenty leeches to the temples—and purgatives persevered in, together with two grains of calomel every three hours. Three days of this plan produced little alteration of the head-ache. A blister was now applied to the nape of the neck—and the leeches reapplied to the temples. Still there was no relief of the symptoms. "The tumefaction of the epigastric and hypochondriac regions, which had existed from the beginning, now rather increased, and much uneasiness was produced on pressure." Twelve leeches were applied to the part. At this period (the 12th day) the boy became comatose, with the eyelids half closed—the pupils insensible to light—paralytic affection of the left arm, thigh, and leg—difficulty of swallowing. In addition to the internal exhibition of calomel, frictions of mercurial ointment were directed to the hypochondriac region, night and morning—cap blister to the head—sinapisms to the feet. In the state above described, the

---

* We can easily conceive that pain might be mitigated by pressure, in this case; but we certainly have much doubt that dyspnœa would be lessened by the application of a compressing force to the abdomen of a dropsical patient.—*Rev.*

† Dr. Henry Fisher. Ed. Journal, Jan. 1825.

little patient lay till the 25th day, during which time the urine and fæces were discharged involuntarily, his pulse ranging from 60 to 66 strokes in the minute—his right hand constantly on the side of his head—left paralytic. On the day above-mentioned, there appeared symptoms of constitutional effects of the mercury, and the pulse rose from 80 to 86—the pupil became slightly sensible to light—stools tinged with bile, and ceasing to exhibit the spinagy appearance. "The alteration really appeared miraculous." "From this period, he recovered rapidly, and in a month was quite well.

We think the above case is entitled to the appellation of hydrocephalus—at least, we have found the pathological characters of the disease in the examination of all those who died with the foregoing symptoms. At the same time, we do not imagine that effusion to any extent had taken place. The case teaches us that we should not too readily despair in the treatment of hydrocephalic children, but persevere till the last. This case also furnishes very strong proof that the green or spinagy stools are not simply the product of the calomel, but a very constant symptom of the hydrocephalic condition of brain. Here the stools changed *during the exhibition of the calomel*, and all the symptoms partook in the contemporary amelioration.

---

16. *Boiling Bath in Chronic Rheumatism.* The following *anceps remedium* we should hardly say was—melius quam nullum. It is recorded in the *Journal Universel des Sciences Med.* for November, 1824.

A young woman had experienced rheumatic pains in her limbs, for six months; the joints, hands, and feet being swollen, and little capable of motion. Leeches, cataplasms, tepid baths, and anodyne embrocations had given but temporary relief. She was advised by a Charlatan to remain in a hot bath for twelve hours—the temperature of which he gradually increased *till it was very near the boiling point!* After six hours sojourn in this torrid zone, the patient lost sensibility—or, at least, recollection, and in the course of another hour was found senseless, her head reclining on a board covering the bath. She was taken out of the water, her face being enormously swollen and blackened—the eye-lids tumefied—the eyes distorted—the skin of a dark red colour—feeling and recollection lost—taciturn delirium—grinding of the teeth—foaming at the mouth—convulsions of the limbs—laborious respiration—distention of the abdomen—hard and irregular pulse.

She was bled from the arm to 28 ounces. She soon recovered recollection and speech—the convulsions ceased, and she only complained of pain at the epigastrium, and thirst. A large emollient cataplasm was applied to the pit of the stomach, and ices were given. She passed a restless night. On the following day, the pain at the epigastrium was very severe, and 40 leeches were applied, which gave much relief. On the third day, the patient complained of pain about the umbilicus, and twenty leeches were applied there, with instant relief. In eight days she was quite recovered. Six weeks afterwards the whole of the epidermis peeled off. Eleven months have now elapsed, and the patient has had no return of the rheumatism.

We think this process bore a tolerable good resemblance to a dip in Medea's Cauldron. The trial was a hazardous one—but appears, in this instance, to have succeeded in curing the original disease.

